# Distinct *daf-16* isoforms regulate specification of vulval precursor cells in *Caenorhabditis elegans*

**DOI:** 10.17912/micropub.biology.000706

**Published:** 2022-12-09

**Authors:** Liberta Cuko, Allison R Cale, Loni Rambo, Macy L Knoblock, Xantha Karp

**Affiliations:** 1 Central Michigan University; 2 Current address: University of Michigan, Ann Arbor

## Abstract

FOXO transcription factors regulate development, longevity, and stress-resistance across species. The *C. elegans FOXO* ortholog, *daf-16,* has three major isoforms with distinct promoters and N-termini. Different combinations of isoforms regulate different processes. Adverse environments can induce dauer diapause after the second larval molt. During dauer, *daf-16* blocks specification of vulval precursor cells, including EGFR/Ras-mediated 1˚ fate specification and LIN-12/Notch-mediated 2˚ fate specification. Using isoform-specific mutants, we find that *daf-16a* and *daf-16f* are functionally redundant for the block to the expression of 1˚ fate markers. In contrast, all three isoforms contribute to blocking the expression of 2˚ fate markers.

**Figure 1. daf-16 isoform-specific mutants express VPC specification markers during dauer:**
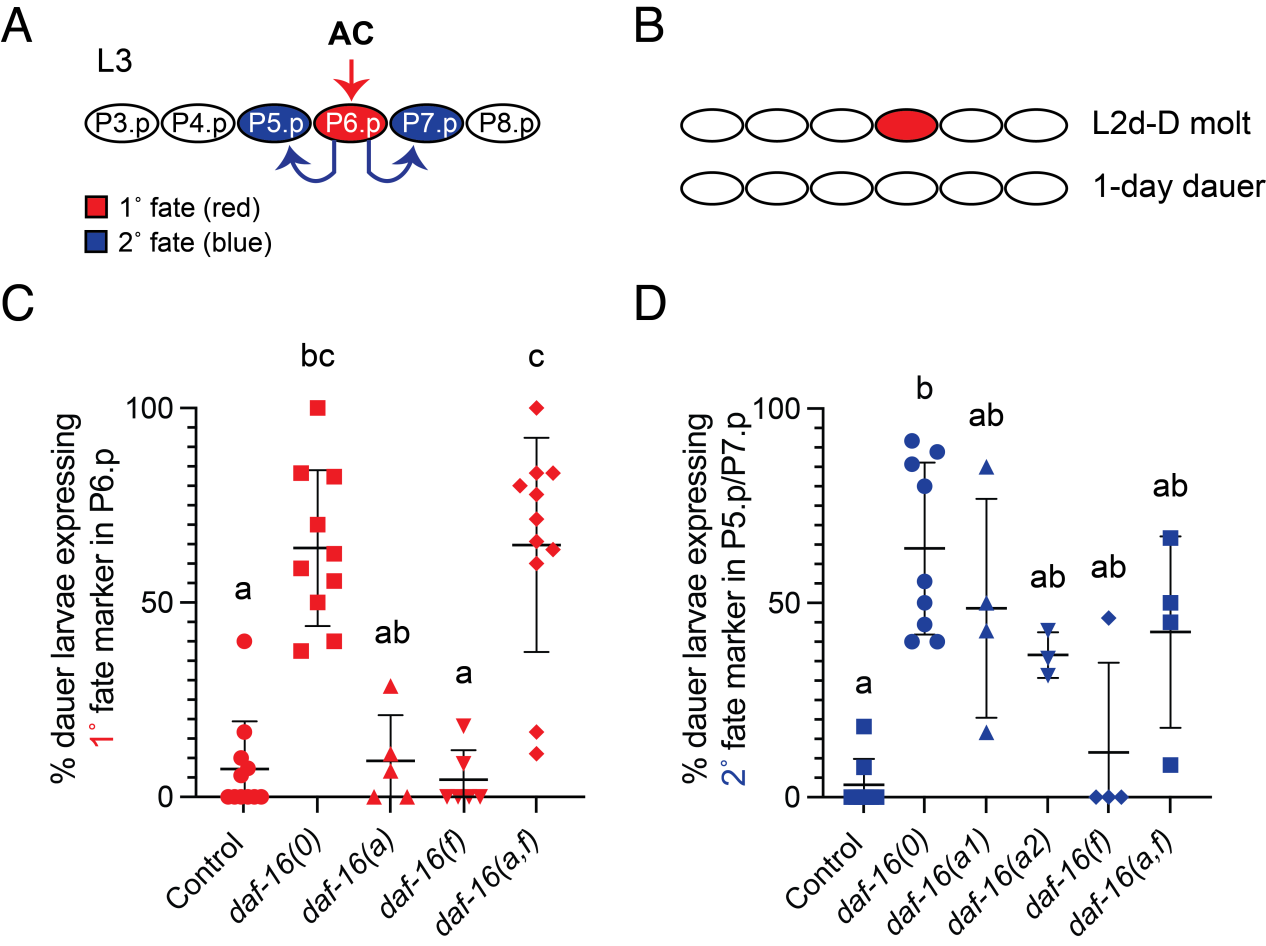
(A) Vulval precursor cell (VPC) specification occurs in the L3 stage during continuous (non-dauer) development (Sternberg 2005). Six equipotential VPCs are named P3.p-P8.p. LIN-3/EGF is secreted from the anchor cell (AC) and a canonical EGFR/Ras/MAPK pathway specifies 1˚ vulval fate in P6.p (red). P6.p then expresses ligands for LIN-12/Notch, activating LIN-12 signaling in the adjacent cells, P5.p and P7.p. LIN-12 signaling specifies 2˚ vulval fate (blue). The remaining VPCs adopt a non-vulval fate (Sternberg 2005). (B) As larvae molt from L2d into dauer (L2d-D molt), 1˚ cell fate marker expression is observed in P6.p (red). However, this expression is lost within one day of entering dauer, such that larvae that have been in the dauer stage for one day (“1-day dauer”) do not express 1˚ fate markers. 2˚ cell fate markers are not observed prior to dauer or during dauer in wild-type or *daf-7* control larvae (Karp and Greenwald 2013). (C-D) The *daf-7(e1372)* allele was used to induce dauer formation and is present in all strains. Strains lacking one or more *daf-16* isoform were scored for the presence or absence of expression of a 1˚ fate marker *(arIs131[lag-2p::yfp])* or 2˚ fate marker *(arIs116[lst-5p::yfp])* . Individual dots represent the percent of larvae expressing the relevant marker in a single trial of 5-36 dauer larvae. The mean and standard deviation for each strain is shown. Letters above the data indicate statistical groupings, where strains that have a letter in common are not statistically different from each other. (Kruskal Wallis with Dunn’s multiple comparison test, α = 0.05). P-values for all non-significant comparisons were > 0.99 with the following exceptions: In panel (C), *daf-16(0)* vs. *daf-16(a)* , p = 0.08. In panel (D), control vs. *daf-16(a1),* p = 0.19. Control vs. *daf-16(a,f),* p = 0.19. *daf-16(0)* vs. *daf-16(f),* p = 0.08. (C) Percent of 1-day dauer larvae that express the *lag-2p::yfp* 1˚ fate marker in P6.p. *daf-16* alleles: *daf-16(0), mgDf50. daf-16(a), tm5030. daf-16(f), tm6659. daf-16(a,f), mg54.* Loss of *daf-16a* and *daf-16f* together recapitulates the phenotype of loss of all *daf-16* isoforms in the null allele *daf-16(0).* (D) Percent of early dauer larvae that express the *lst-5p::yfp* 2˚ fate marker in P5.p and/or P7.p. *daf-16* alleles: *daf-16(0), mgDf50. daf-16(a1), tm5030. daf-16(a2), tm5032. daf-16(f), tm6659. daf-16(a,f), mg54.* Loss of each *daf-16* isoform tested produces a phenotype intermediate between the control *daf-* 7 strain and the *daf-16(0)* allele *.*

## Description

Forkhead box O (FOXO) transcription factors regulate critical processes including development, metabolism, longevity, and stress-resistance. The *C. elegans FOXO* ortholog, *daf-16,* has been foundational for the discovery of many of these roles which are conserved across species (Kenyon et al. 1993; Murphy and Hu 2013; Tissenbaum 2018). *daf-16* was discovered for its role in promoting entry into dauer diapause which occurs after the second larval molt in response to unfavorable environments (Cassada and Russell 1975; Vowels and Thomas 1992).

There are three major isoforms of *daf-16: daf-16a, daf-16b,* and *daf-16d/f/h* (hereafter called *“daf-16f”),* each with its own promoter (see Methods). These isoforms are expressed in distinct tissues and produce proteins that differ at the N-terminus (Lin et al. 1997; Ogg et al. 1997; Kwon et al. 2010) *.* Of the three isoforms, *daf-16b* differs the most from the other two: while *daf-16a* and *daf-16f* are broadly expressed, *daf-16b* expression is found primarily in neurons, the pharynx, and somatic gonad. Furthermore, DAF-16a and DAF-16f have more amino acids in common, including the entire DNA-binding domain, whereas a portion of the DAF-16b DNA-binding domain comes from a distinct exon (Murphy and Hu 2013).

The molecular differences between isoforms appear to correlate with their biological functions. *daf-16a* and *daf-16f* together regulate longevity, dauer formation, and stress-resistance, with little to no input from *daf-16b* (Lee et al. 2001; Kwon et al. 2010; Chen et al. 2015). In contrast, *daf-16b* regulates neuronal morphology and behavior (Christensen et al. 2011; Sun et al. 2019). However, there are some examples of shared roles between *daf-16b* and other isoforms. *daf-16a* and *daf-16b* both contribute to innate immunity in adults, though *daf-16a* plays the predominant role (McHugh et al. 2020). Finally, all three isoforms contribute to preventing expression of an adult cell fate marker in the hypodermis of dauer larvae (Wirick et al. 2021).

During dauer, *daf-16* is part of a mechanism to block specification of vulval precursor cells (VPCs) (Karp and Greenwald 2013). Six VPCs, P3.p-P8.p, are born in the L1 stage, remain multipotent in the L2 stage, and are specified to one of two vulval fates in the L3 stage. LIN-3/EGF secreted from the anchor cell specifies 1˚ fate in the underlying VPC, P6.p. P6.p then signals to its neighbors via LIN-12/Notch to specify 2˚ cell fate. VPCs that do not receive either signal take on a non-vulval fate (3˚) (Fig. 1A) (Sternberg 2005). In the dauer life history, 1˚ fate specification begins prior to dauer formation, but this specification is erased within one day and thereafter dauer larvae do not express either 1˚ or 2˚ cell fate markers (Fig. 1B) (Karp and Greenwald 2013). The block to 1˚ fate occurs at or upstream of Ras activation, whereas the block to 2˚ fate appears to occur downstream or in parallel to activation of LIN-12/Notch (Karp and Greenwald 2013; O’Keeffe and Greenwald 2022). However, dauer larvae with a null allele of *daf-16 (daf-16(0))* express both 1˚ and 2˚ markers during dauer, indicating that *daf-16* opposes both 1˚ and 2˚ cell fate specification (Karp and Greenwald 2013).

To determine which *daf-16* isoforms are responsible for blocking VPC specification during dauer, we took advantage of existing isoform-specific mutants. These mutations eliminate either *daf-16a, daf-16f,* or both *daf-16a* and *daf-16f (daf-16a,f),* without affecting the expression of the remaining isoforms (Chen et al. 2015). Specifically, the *daf-16f* allele, *tm6659,* deletes the exons specific to the *daf-16f* isoform. Two similar alleles, *tm5030* and *tm5032,* denoted here as *daf-16(a1)* and *daf-16(a2),* each delete a portion of the *daf-16a-* specific exon and cause frameshift mutations. Finally, the *daf-16(a,f)* allele, *mg54,* is a nonsense mutation in an exon that is common to the *daf-16f* and *daf-16a* isoforms but upstream of the first *daf-16b* exon (Chen et al. 2015). We crossed these alleles into strains containing either 1˚ or 2˚ cell fate markers. All strains also contained the *daf-7(e1372)* mutation to induce dauer formation in *daf-16* mutants (Vowels and Thomas 1992; Larsen et al. 1995; Karp 2018).

To determine which *daf-16* isoforms are involved in blocking 1˚ fate specification, we used *lag-2p::yfp* as our 1˚ fate marker. *lag-2* encodes a LIN-12 ligand expressed by P6.p after commitment to 1˚ fate (Chen and Greenwald 2004; Zhang and Greenwald 2011). Consistent with prior work, in control *daf-7* dauer larvae, *lag-2p::yfp* was expressed as larvae molt into dauer, but expression faded over the first day in dauer as multipotency was re-established. In contrast, *daf-16(0); daf-7* larvae continued to express *lag-2p::yfp* at high penetrance (Fig. 1C) (Karp and Greenwald 2013). While loss of *daf-16a* caused only minimal *lag-2p::yfp* expression in 1-day dauer larvae and loss of *daf-16f* was not different from the control *daf-7* strain, loss of both isoforms together produced a phenotype statistically indistinguishable from the null allele (Fig. 1C). With the caveat that we were not able to directly test a *daf-16(b)* allele, these data suggest that isoforms *a* and *f* function together to block 1˚ specification and that *daf-16b* plays at most a minor role in this process. This role is similar to the requirement for those isoforms to regulate dauer formation, lifespan, and stress resistance (Lee et al. 2001; Kwon et al. 2010; Chen et al. 2015).

To determine which *daf-16* isoforms are involved in blocking 2˚ fate specification, we used *lst-5::yfp* as our 2˚ marker. *lst-5* is a direct transcriptional target of LIN-12 and is expressed in P5.p and P7.p after commitment to 2˚ fate (Li and Greenwald 2010; Underwood et al. 2017). Control *daf-7* larvae that had recently molted into dauer expressed very little *lst-5p::yfp* in P5p or P7.p. In contrast, *daf-16(0); daf-7* larvae displayed penetrant *lst-5p::yfp* expression at this time (Fig. 1D) (Karp and Greenwald 2013). Loss of *daf-16a, daf-16f,* or both *daf-16a* and *daf-16f* produced an intermediate phenotype such that the penetrance of *lst-5p::yfp* expression was not statistically different from either the *daf-7* or the *daf-16(0); daf-7* controls (Fig. 1D). These data suggest that all three isoforms contribute to blocking 2˚ cell fate specification. This role is similar to the requirement for all three isoforms to block expression of the *col-19p::gfp* adult cell fate marker in the lateral hypodermis of dauer larvae (Wirick et al. 2021).

Taken together, we have found distinct requirements for *daf-16* isoforms to block 1˚ and 2˚ cell fate specification during dauer. Promoter swap experiments indicate that differences in expression explain requirements for different isoforms for the regulation of lifespan and dauer formation (Lee et al. 2001; Kwon et al. 2010). *daf-16* is required in VPCs to block expression of the 1˚ fate marker used in this study; the focus of *daf-16* activity for its role in 2˚ fate is unknown (Karp and Greenwald 2013). Furthermore, in wild-type larvae, *lag-2p::yfp* is expressed during the L2d-dauer molt and downregulated during dauer whereas *lst-5p::yfp* is not expressed in the L2d-dauer molt or dauer (Karp and Greenwald 2013). This difference in expression raises the possibility that the block to 1˚ fate specification occurs later than the block to 2˚ specification. It will be interesting to investigate whether the distinct requirements identified in this study are due to differences in expression of *daf-16* isoforms, either spatially or temporally, differences in the focus of *daf-16* activity for 1˚ and 2˚ fate, or the unique N-terminus of each *daf-16* isoform.

## Methods

**Strains and maintenance**

*C. elegans* strains were maintained at 20˚C on NGM plates with *E. coli* strain OP50 as a food source.

**Dauer induction**

The *daf-7(e1372)* allele was present in all strains and used to induce dauer formation (Vowels and Thomas 1992; Larsen et al. 1995; Karp 2018). Ten to thirty gravid adult hermaphrodites of the desired strains were added to seeded plates at 24˚C or 25˚C. The adults were allowed to lay eggs for 5 hours at 25˚C. The adults were removed, and the embryos were incubated at 25˚C for a further 48 hours for “early dauers” or 72 hours for “1-day dauers”. At 25˚C, *daf-7* larvae molt into dauer at approximately 48 hours after egg-laying (Karp 2018). To ensure larvae were within the dauer stage, prior to scoring, putative dauer larvae were added to an NGM plate seeded with 10x concentrated OP50 mixed with red fluorescent beads (Sigma L3280) at a 1:1000 ratio (v/v) of beads to 10x OP50. Larvae were incubated on bead-containing plates for 30 minutes. Dauer larvae, including *daf-16(0)* dauer larvae, do not ingest food and thus do not take up any fluorescent beads during this time (Nika et al. 2016); therefore, larvae that lacked beads were used for experiments. To ensure larvae were not still within the L2d-dauer molt, DIC optics were used to confirm radial constriction and fully formed dauer alae.

**Microscopy**

Larvae were anesthetized in 0.1M levamisole on 2% agarose pads. Worms were imaged using a Zeiss AxioImager D2 compound microscope with a 63x objective, an AxioCam MRm Rev 3 camera, and Zen software (Zeiss). To avoid bleaching, DIC optics were used to locate the relevant VPCs followed by snapping a single image of DIC and YFP channels using a 500ms exposure time for YFP. Images were categorized as on or off, where on indicated detectable YFP expression in the relevant VPCs in the image. Controls were performed in parallel with isoform-specific mutants, and each strain was scored by 2-3 independent researchers. The combined data from all researchers are shown in Figure 1.

**Statistics**

The percent of dauer larvae expressing the relevant marker in each individual trial was treated as an individual data point. For each strain, 3-11 trials were performed, where each trial consisted of 5-36 dauer larvae. Differences between strains were assessed using Kruskal Wallis with a Dunn’s multiple comparison test (α = 0.05). The total number of worms scored for each strain was: GS5620, *n=* 142; GS5997, *n=* 123; XV96, *n=* 82; XV95, *n=* 93; XV97, *n=* 130; GS6106, *n=* 64; GS6163, *n=* 80; XV92, *n=* 51; XV93, *n=* 37; XV91, *n=* 53; XV90, *n=* 54.

***daf-16* isoforms**

The *daf-16* isoforms described in this study are those that have been defined and characterized in the literature (Lin et al. 1997; Ogg et al. 1997; Lee et al. 2001; Kwon et al. 2010; Chen et al. 2015). All of these isoforms contain a Forkhead DNA binding domain, phosphorylation sites for AKT, and N-terminal phosphorylation sites. However, the sequences at the N-terminus vary between isoforms (reviewed in Murphy and Hu 2013). In the current version of WormBase (WS286), there are 11 *daf-16* isoforms listed. The isoform described as *daf-16a* in the literature corresponds to the nearly identical R13H8.1b.1 and R13H8.1c.1 isoforms on WormBase. The isoform described as *daf-16b* in the literature corresponds to R13H8.1a.1 on WormBase. The isoforms described as *daf-16d/f/h* in the literature correspond to isoforms R13H8.1d.1, R13H8.1f.1, R13H8.1h.1, R13H8.1i.1, and R13H8.1k.1. In addition to these isoforms, WormBase also lists R13H8.1e.1, R13H8.1l.1, and R13H8.1m.1, none of which have been characterized in the literature. R13H8.1e.1 lacks a DNA binding domain. R13H8.1l.1 and R13H8.1m.1 are predicted to encode the complete Forkhead DNA binding domain but lack the N-terminal phosphorylation sites. The *daf-16(a)* and *daf-16(f)* mutations, *tm5030, tm5032,* and *tm6659* would not affect any of these uncharacterized isoforms (Chen et al. 2015). However, the *daf-16(a,f)* mutation, *mg54,* is an early stop codon that should disrupt isoforms R13H8.1l.1 and R13H8.1m.1 (Ogg et al. 1997).

## Reagents

**Table:** 

**Strain name**	**Genotype**	***daf-16* isoforms deleted **	**Reference for strain**
GS5620	*daf-7(e1372) arIs131 [lag-2p::2xNLS::YFP + ceh-22p::GFP + pha-1(+)]*	None (wild type)	Karp & Greenwald 2013
GS5997	*daf-16(mgDf50); daf-7(e1372) arIs131*	All (null allele)	Karp & Greenwald 2013
XV96	*daf-16(tm5030); daf-7(e1372) arIs131*	*daf-16a*	This work
XV95	*daf-16(tm6659); daf-7(e1372) arIs131*	*daf-16f*	This work
XV97	*daf-16(mg54); daf-7(e1372) arIs131*	*daf-16a,f*	This work
GS6106	*daf-7(e1372); arIs116 [lst-5p::2xNLS::YFP + ttx-3p::GFP + pha-1(+)]*	None (wild type)	Karp & Greenwald 2013
GS6163	*daf-16(mgDf50); daf-7(e1372); arIs116*	All (null allele)	Karp & Greenwald 2013
XV92	*daf-16(tm5030); daf-7(e1372); arIs116*	*daf-16a*	This work
XV93	*daf-16(tm5032); daf-7(e1372); arIs116*	*daf-16a*	This work
XV91	*daf-16(tm6659); daf-7(e1372); arIs116*	*daf-16f*	This work
XV90	*daf-16(mg54); daf-7(e1372); arIs116*	*daf-16a,f*	This work
